# Quality of life impact of eye diseases: a Save Sight Registries study

**DOI:** 10.1111/ceo.14050

**Published:** 2022-02-07

**Authors:** Himal Kandel, Vuong Nguyen, Stefano Piermarocchi, Lala Ceklic, Kelvin Teo, Francisco Arnalich‐Montiel, Stefania Miotto, Vincent Daien, Mark C. Gillies, Stephanie L. Watson

**Affiliations:** ^1^ Save Sight Institute, Faculty of Medicine and Health The University of Sydney Sydney New South Wales Australia; ^2^ Department of Ophthalmology Padua‐Camposampiero Hospital Padua Italy; ^3^ Centar za zastitu vida" Pale Eastern Sarajevo Bosnia and Herzegovina; ^4^ Singapore National Eye Centre Singapore Eye Research Institute Singapore Singapore; ^5^ Cornea & External Eye Diseases, Hospital Universitario Ramón y Cajal IRYCIS Madrid Spain; ^6^ Ophthalmology University Hospital Montpellier Montpellier France

**Keywords:** age‐related macular degeneration, diabetic macular edema, emotional, keratoconus, patient‐reported outcomes, Quality of life, Questionnaire, Rasch analysis, Retinal vein occlusion, Visual function

## Abstract

**Background:**

The objectives of this study were to evaluate the quality‐of‐life (QoL) impact of eye diseases (keratoconus; neovascular age‐related macular degeneration, AMD; retinal vein occlusion, RVO; and diabetic macular edema, DME) using the Impact of Vision Impairment (IVI) questionnaire, and to determine the relationship between the IVI scores and visual acuity.

**Methods:**

This cross‐sectional, multicentre, real‐world study utilised the prospective, web‐based Save Sight Registries. The IVI was completed by 1557 patients: 307 with keratoconus, 1049 with AMD, 148 with RVO and 53 with DME. Statistical analysis included Rasch analysis, Welch *t*‐test, one‐way ANOVA, Tukey's test, Pearson correlation, and multiple regression.

**Results:**

The IVI scales (Overall; Visual Function, VF; Emotional, EM) had robust psychometric properties. The keratoconus patients had the worst Overall (adjusted mean: 48.2 vs. DME 58.8, RVO 64.6, AMD 67.6 units), VF (47.7 vs. DME 59.4, RVO 65.9, AMD 68.9 units) and EM (50.8 vs. DME 63.1, RVO 69.2, AMD 71.8 units) scores (all *p* < 0.05).

The IVI scales scores weakly correlated with better and worse eye visual acuity (Pearson's *r* 0.24–0.39, all *p* < 0.05). The correlations were similar in the better eye (Overall 0.35, VF 0.39, EM 0.24) and the worse eye (Overall 0.31, VF 0.33, EM 0.25) visual acuity. Correlations with visual acuity were stronger for VF than for the EM scores.

**Conclusions:**

The IVI was a psychometrically robust QoL questionnaire. Keratoconus patients had worse IVI scores than patients with retinal diseases. The low strength of correlations between visual acuity and QoL scores, although statistically significant, suggested that a complex relationship exists.

## 1 INTRODUCTION

Eye diseases may lead to visual impairment which is one of the public's most feared disabilities.^1^ Visual impairment is often linked to falls, injury, hip fractures, depression, loss of independence, inability to self‐care, fear of blindness, the early need of nursing home placement and overall a significant decline in quality of life (QoL).^2^, ^3^, ^4^, ^5^, ^6^, ^7^ Traditionally, clinical measures such as visual acuity have been the main outcome measures in ophthalmology. However, clinical measures do not capture the overall impact of the diseases and their treatments on patients' visual functioning, emotional well‐being and QoL.^8^, ^9^, ^10^, ^11^ The ultimate aim of eye care, like any other health care, is to address the QoL impact of eye diseases.^12^ QoL impact of eye disease can be evaluated using patient‐reported outcome (PRO) measures that capture patient perspectives.^13^, ^14^

PRO measures are now increasingly used as an essential component of a patient‐centred health system,^15^ particularly to comprehensively assess the impact of disease and efficacy of treatments on patients.^16^, ^17^ The popularity of PRO measures for research and clinical purposes is likely to grow as regulatory bodies such as the US Food and Drug Administration recommend their use for determining product effectiveness.^18^, ^19^ While there has been a rapid expansion of the literature on ophthalmic QoL, there are a limited number of comparable studies with identical methods. The comparative evidence on the relative impact of eye diseases on QoL is largely inconclusive (Table 1).^7^, ^10^, ^20^, ^21^, ^22^, ^23^, ^24^, ^25^ In most cases, the QoL‐evaluation has been conducted in research settings^10^, ^20^, ^21^, ^22^, ^23^, ^24^ and the applicability of the findings in real‐world routine clinical practice is not well understood.

**TABLE 1:** Existing evidence on the comparative impact of eye diseases on people's quality‐of‐life

Study (Author year; Location; Population; Ocular morbidity)	Measure	Key finding
Finger 2011^7^; Germany; hospital‐based; AMD 54, DR 27, Glaucoma 15, Other retinal diseases 49, Corneal disease 12, Others 27	Impact of vision impairment (IVI)	There were no statistically significant differences in the Functional and Emotional IVI scores between eye diseases including AMD, DR and corneal diseases when adjusted for visual acuity. People with DR had the worst IVI scores followed by AMD, corneal diseases and glaucoma, respectively.
Fenwick 2016 [Singapore Chinese Eye Study]^20^; Singapore; Population‐based; URE 377, Cataract 244, AMD 19, DR 14, Glaucoma 10, Others 54	IVI‐Mobility and Independence items	People with glaucoma had the worst Mobility and Independence scores followed by DR, Cataract, AMD and URE, respectively.
Fenwick 2017 [Singapore Chinese Eye Study]^21^; Singapore; Population‐based; URE 377, Cataract 244, AMD 19, DR 14, Glaucoma 10, Others 54	IVI‐emotional items	People with glaucoma had the worst emotional status followed by DR, AMD, Cataract and URE, respectively.
Lamoureux 2008 [Singapore Malay Eye Study]^22^; Singapore; Population‐based; URE 670, Cataract 447, DR 31, AMD 22, Glaucoma 21, Others 56	Visual functioning‐11 (VF‐11)	People with glaucoma had the worst visual functioning scores followed by AMD, DR, Cataract and URE, respectively.
Nutheti 2007 [Andhra Pradesh Eye Disease Study]^10^; India; Population‐based; Cataract 760, Refractive errors 525, Glaucoma 41, Retinal disease 84, Corneal disease 60	Unnamed quality of life questionnaire	Patients with corneal and retinal diseases had worse visual function scores than those with a cataract.
Broman 2002 [Proyecto VER]^23^; USA; Population‐based; URE 405, Cataract 374, DR 290, Glaucoma 100, Others 29	National Eye Institute Visual Function Questionnaire (NEI‐VFQ‐25)	In general, people with DR and Glaucoma had worse scores than cataract or uncorrected refractive error. People with glaucoma had worse NEI‐VFQ subscales scores than the people with DR except for the General Health subscale (i.e., 12 out of 13 subscales).
Chia 2004 [Blue Mountains Eye Study]^24^; Australia; Population‐based; Cataract 539, AMD 99, Cataract and AMD 82	Short Form‐36 (SF‐36)	People with AMD had slightly lower physical and mental component scores than people with a cataract. The authors concluded that the quality‐of‐life status was related to the severity of visual impairment but not to the underlying eye disease.
Brown 2002^25^; USA; hospital‐based; DR 333, AMD 246	Time trade‐off (utility)	The health utility values for patients with DR and AMD were similar when stratified by visual acuity levels. Overall, DR patients had slightly higher utility than AMD patients.

Abbreviations: AMD, age‐related macular degeneration; DR, diabetic retinopathy; URE, uncorrected refractive error.

The Impact of Vision Impairment (IVI) questionnaire is a widely used, high‐quality and International Consortium for Health Outcomes Measurement (ICHOM) recommended non‐disease‐specific ophthalmic PRO measure.^26^, ^27^, ^28^ The use of IVI to compare the QoL impact of eye conditions may help clinicians and researchers understand the disease impacts and inform evidence‐based resource allocations at the planning level. The primary objective of this study was to evaluate the QoL impact of anterior (keratoconus) and posterior (neovascular age‐related macular degeneration, AMD; retinal vein occlusion, RVO; and diabetic macular edema, DME) eye diseases using the IVI. The secondary objectives were to evaluate the psychometric properties and validity of the IVI questionnaire in routine clinical practice, and to determine the relationship between the IVI scores and visual acuity.

## 2 METHODS

This descriptive, cross‐sectional study was conducted using the prospectively designed SSR database (http://savesightregistries.org/). The SSR is a multi‐national web‐based database of patients with eye diseases that allows routine collection of clinical and patient‐reported data and monitoring of outcomes over time.^29^, ^30^, ^31^, ^32^ The current study included the participants from 44 practices with an established diagnosis of keratoconus, AMD, RVO or DME in one or both eyes who self‐administered the IVI questionnaire. The clinicians who use the Save Sight Registries in their routine clinical practice collected the IVI data. The IVI questionnaires were interviewer‐ or self‐administered in the English language. Patients enrolled in the Save Sight Registries were eligible and were given as much time as they needed to answer the IVI questions. Patients made their own decisions without the contribution of their families or friends. Similarly, the patients with multiple ocular conditions that could impact QoL (e.g., a patient with keratoconus and cataract) were excluded. Patients with cognitive difficulty were also excluded. For patients with more than one IVI visit, the baseline visit was considered.

Ethics clearance approvals in Australia were obtained from the Sydney Local Health District HREC for public hospitals and the ethics committee of the Royal Australian and New Zealand College of Ophthalmologists for the private sites. The international centres obtained approvals from the relevant local ethics committees. Informed consent was obtained from each patient. The data were de‐identified before analysis.

### 2.1 Statistical analysis

Rasch analysis on the IVI data (see Appendix S1, Supplementary Table S1.5 for IVI content) was conducted using the Winsteps software (Version 3.92.1; Winsteps, Chicago, IL) to evaluate the psychometric properties of the IVI scales and obtain scores (person measures) on Overall, Visual function (VF) and Emotional (EM) domains. We utilised the Andrich Rating Scale model of Rasch analysis and followed methods as in our previously published study^33^ and further details are also provided in Appendix S1. In brief, the Rasch model is a probabilistic model which converts categorical PRO data into interval‐level data by log‐transformation. It aligns persons and items on a common invariant interval‐level scale. The Rasch model has its roots in educational testing and is based on the principle that a person with higher ability is more likely to be successful in answering a question, and the easier questions are more likely to be answered correctly.^34^, ^35^, ^36^ Rasch analysis addresses the serious flaws of the traditional summary scoring methods which consider each item in a PRO measurement scale as of the same value and each response option as equally separated from the adjacent option.^37^ The IVI scores were scaled using the UScale command on Winsteps such that the scores were between 0 and 100, and the item mean was placed at 50 units, for convenience.

The Rasch parameters evaluated included response category functioning, unidimensionality, fit statistics, measurement precision, targeting and differential item functioning. A detailed discussion on the Rasch parameters is beyond the scope of this study and the readers are encouraged to refer to published literature elsewhere.^27^, ^33^, ^34^, ^36^ The significance of each parameter investigated in this study are briefly described below.

The response categories were evaluated to ensure the thresholds advanced monotonically with uniform spacing between the response options. The dimensionality was assessed using principal component analysis (PCA) of the residuals to ensure each IVI scale measured only one underlying construct which is a fundamental property of measurement. On a unidimensional scale, most of the variance is explained by the principal factor and the Eigenvalue of the first contrast is less than 3.0. Similarly, the fit statistics indicated how well the data met the Rasch model expectations. The infit and outfit mean square (MnSq) less than 1.3 is desired which indicates the presence of less than 30% excess variance in the data than the Rasch model expectation. When item misfits were observed, person‐weighting was done such that persons with erratic responses (residuals ≥ |4|) were weighted zero to stop their influence on fit statistics or measures of other persons or items. Similarly, measurement precision was determined by the person reliability or person separation index. The person separation index >2.0 or person‐reliability >0.80 indicated that the IVI scales could differentiate between persons with three levels of latent traits (i.e., three levels of Overall, VF and EM) measured. Likewise, item separation index >3.0 (reliability, 0.90) was considered necessary for achieving reliable item hierarchy. Targeting informed whether the persons and items had a similar spread in the common scale, and a low difference between item and person means indicated good targeting.^33^ Finally, item bias, also known as differential item functioning (DIF) was assessed to ensure different participant groups by age (≤68 vs. >68 years, based on mean), gender, country, visual acuity (<70 vs. ≥70 logMAR letters) and eye disease (corneal vs. retinal). A DIF size >4.5 units (i.e., 0.64 logits) with corresponding *p*‐value <0.05 was considered a notable DIF.^38^

Other statistical analyses were performed using R Statistical Software (Version 4.0.2). Comparisons of IVI scores between demographic and clinical subgroups were assessed using Welch's *t*‐test for two groups or one‐way analysis of variance (ANOVA) followed by Tukey's test for pairwise evaluation. Pearson's correlation (r) was used to determine the associations between the IVI scores and visual acuity. Multiple regression was carried out with IVI scores as the outcome variables, and visual acuity, age, gender, eye disease as the exposure variables, and the adjusted IVI scores were obtained. A *p*‐value <0.05 was considered statistically significant. The relationships between clinical factors such as disease severity with the IVI scores were evaluated considering the better eye unless otherwise specified as the IVI scores had slightly higher correlations with the better eye visual acuity than with worse eye visual acuity in the preliminary analysis.^39^

## 3 RESULTS

The IVI was completed by 1557 patients: 307 with keratoconus, 1049 with AMD, 148 with RVO and 53 with DME (Table 2). The mean ± SD age of the participants was 68.1 ± 22.1 (range 11–100) years. 767 (49.2%) of the participants were female. The patients were from six countries—Australia, Singapore, Italy, Bosnia and Herzegovina, Spain and France; 83.2% of them were Australian and 74.6% were White or Caucasians. A majority (78.6%) of the patients had received treatments in their better eye: anti‐VEGF or laser for retinal conditions (AMD, 984; DME, 47; RVO, 149) and corneal cross‐linking (*n* = 44) for keratoconus. More than half (844, 54.1%; AMD 723, Keratoconus 10, DME 37, RVO 74) patients completed the IVI within one week of starting treatment.

**TABLE 2:** Demographic and clinical characteristics of the participants

*N*	1518
Age, years	
Mean (SD)	68.1 (22.1)
Min, max	11, 100
Gender, *n* (%)	
Female	767 (49.2)
Male	791 (50.8)
Country of residence, *n* (%)	
Australia	1296 (83.2)
Singapore	212 (13.6)
Others	50 (3.2)
Ethnicity, *n* (%)	
White or Caucasian	1162 (74.6)
Asian	276 (17.7)
Others	41 (2.6)
Unspecified	79 (5.1)
Visual acuity—overall, Mean (SD) logMAR letters	
Better eye	71.5 (14.6)
Worse eye	54.2 (22.3)
Visual acuity—By eye disease, Mean (SD) logMAR letters	
Keratoconus	75.3 (14.1)
Age related macular degeneration	56.2 (21.2)
Retinal Vein Occlusion	60.1 (21.4)
Diabetic macular edema	73.8 (12.9)
Keratoconus, *n* (%)	
Mild	92 (30.0)
Moderate	115 (38.8)
Severe	77 (25.1)
Missing *K*_max_	23 (7.5)
Total	307 (100)
Age related macular degeneration (*N* = 1049)	
Angiographic lesion type, *n* (%)	
Type 1 (Occult)	544 (51.9)
Type 2 (Classic)	171 (18.2)
Type 3 (Retinal Angiomatous Proliferation)	31 (3.0)
Idiopathic Polypoidal Choroidal Vasculopathy (IPCV)	136 (13.0)
Juxtapapillary	10 (1.0)
Disciform scar	13 (1.2)
Unclassified/missing data	144 (13.7)
Geographic atrophy, n (%)	
None	388 (37.0)
Sub‐foveal	36 (3.4)
Extrafoveal	63 (6.7)
Unclassified/not assessed	562 (53.6)
Retinal vein occlusion, *n* (%)	
BRVO	45 (30.4)
CRVO	95 (64.2)
HRVO	8 (5.4)
Total	148 (100)
Diabetic macular edema (*N* = 53)	
Diabetic retinopathy grading, n (%)	
Mild NPDR	14 (26.4)
Moderate NPDR	20 (37.7)
Severe NPDR	11 (20.8)
PDR High risk	3 (5.7)
PDR non‐high risk	1 (1.9)
Treated PDR	4 (7.5)
DME activity, *n* (%)	
Centre involving CSME, %	42 (79.2)
Non‐centre involving CSME, %	4 (7.5)
No CSME, %	7 (13.2)

Abbreviations: AMD, age‐related macular degeneration; BRVO, branched‐retinal vein occlusion; CRVO, central retinal vein occlusion (CRVO); CSME, clinically significant macular edema; DME, diabetic macular edema; HRVO, hemi‐retinal vein occlusion; logMAR, logarithm of minimal angle of resolution; NPDR, non‐proliferative diabetic retinopathy; PDR, proliferative diabetic retinopathy.

### 3.1 Psychometric properties of the IVI scales

**FIGURE 1:**
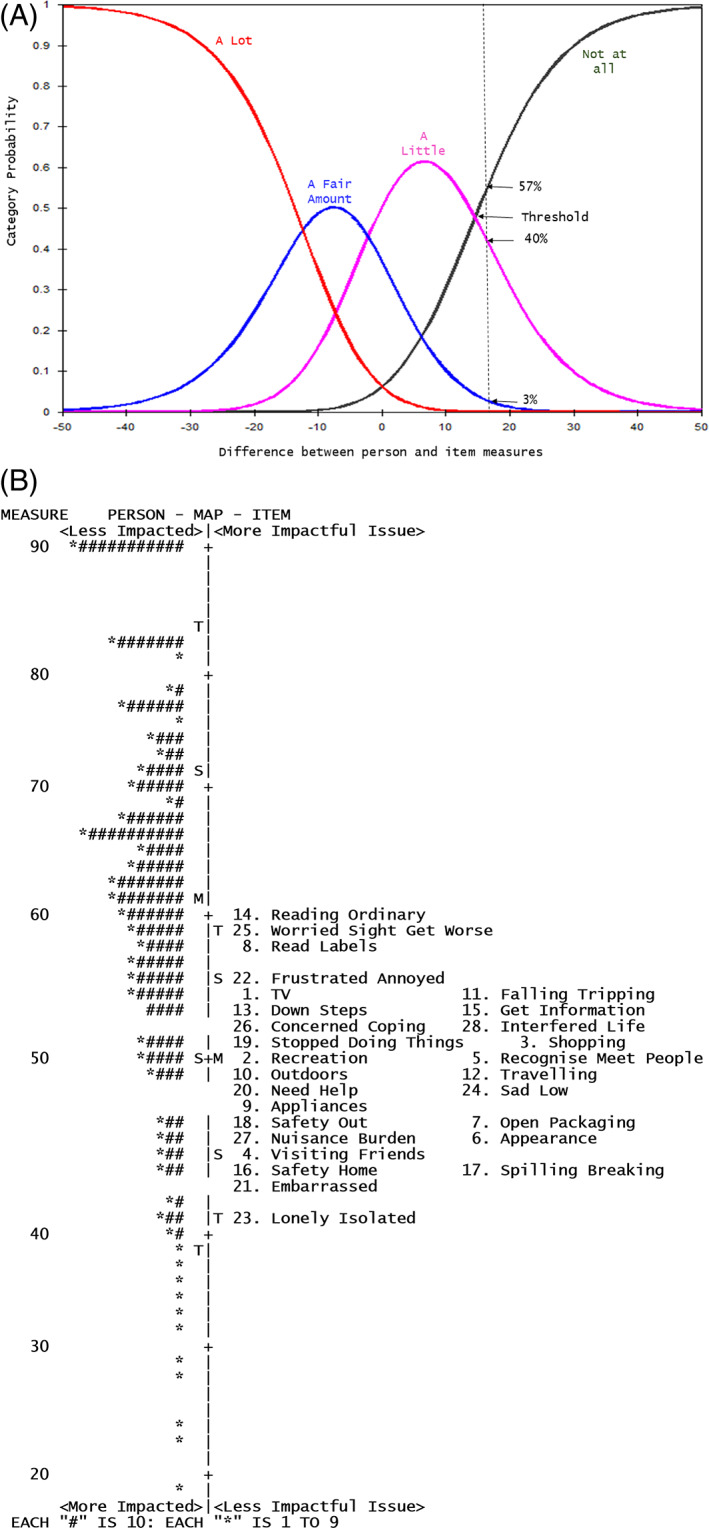
(A) Category probability curves for the Impact of Vision Impairment (IVI)—Emotional scale items. At the location of the dotted line (as an example), the difference between person and item measure is 17 units. At this point of the continuum, the probabilities of endorsing ‘A lot’, ‘A fair amount’, ‘A little’ and ‘Not at all’ are 0%, 3%, 40% and 57% respectively. At a threshold, the probability of endorsing two response options is equal. (B) Person‐Item map for the Impact of Vision Impairment (IVI)‐Overall scale. Persons are located in the left, with their abilities in the latent traits from low (at the bottom) to high (at the top). Items are placed at the right side with their difficulty level of the latent trait from low (bottom) to high (top). *M* = mean, *S* = one standard deviation from the mean, *T* = two standard deviations from the mean

The IVI scales (Overall, VF and EM) had satisfactory psychometric properties including ordered response categories (Figure 1A) with good spacing between the thresholds and no floor or ceiling effects. The Overall IVI scale was essentially unidimensional based on high‐PCA variance explained by the measure, high ratio of the variance explained by the items to the variance explained by the first contrast, a high correlation between the first and second clusters, and good fit statistics. The eigenvalue of the first contrast (3.4) suggested the possibility of forming subscales which eventually resulted in the formation of VF and EM scales (Appendix S1). Measurement precision for VF and EM scales were similar (PSI, VF 2.07 vs. EM 2.12). The variance explained by the measure was higher for EM (68.1%) than for VF (57.3%). The IVI scales had satisfactory fit statistics (MnSq 0.71 to 1.37) and no notable differential item functioning by age, gender, country, visual acuity and eye disease (Table 3). The person‐item map (Figure 1B) shows the relative location of persons and items in a common linear scale. For example, the reading items were the most impactful VF items and concern about eyesight getting worse was the most impactful EM issue for this population. A difference of 6.58 units was found to be the minimally important difference (MID): the half standard deviation of the mean person measure for the Overall IVI scale.^40^

**TABLE 3:** Key Psychometric properties of the IVI scales

Parameters	Overall	Visual function (VF)	Emotional (EM)
No. of items	28	20	8
No. of people	1557	1557	1557
Response categories	Ordered, well‐spaced	Ordered, well‐spaced	Ordered, well‐spaced
Person separation index, Person reliability	2.54, 0.87	2.07, 0.81	2.12, 0.82
Item separation index, Item reliability	13.66, 0.99	12.63, 0.99	20.55, 1.0
PCA, variance explained by the measure	56.9%	57.3%	68.1%
PCA, eigen value for the first contrast (% unexplained variance in first contrast)	3.4 (5.3%)	2.4 (5.2%)	1.6 (6.5%)
Items with item infit (MnSq) > 1.30	‐	‐	EM21: 1.35
Items with outfit (MnSq) > 1.30	EM25: 1.37	‐	‐
Targeting (Person mean—Item mean)	13.23 units (1.89 logits)	14.17 units (2.02 logits)	17.16 units (2.45 logits)
Measurement range, logits scaled 7X	41.34–59.76 units	43.27–60.65 units	37.10–64.17 units

Abbreviation: PCA, principal component analysis.

### 3.2 Evaluation of the quality‐of‐life impact

The higher IVI scores (Overall, VF and EM) represented less impact from eye disease (better visual functioning, emotional well‐being and overall QoL). The correlation of Overall and EM scale scores with age was weak but statistically significant (Spearman's *r*: Overall: −0.08 and EM −0.14; both *p* < 0.05), indicating worse QoL status with increasing age. However, the correlation of the VF score with age was not significant (Spearman's *r*, 0.04; *p* = 0.093).

**FIGURE 2:**
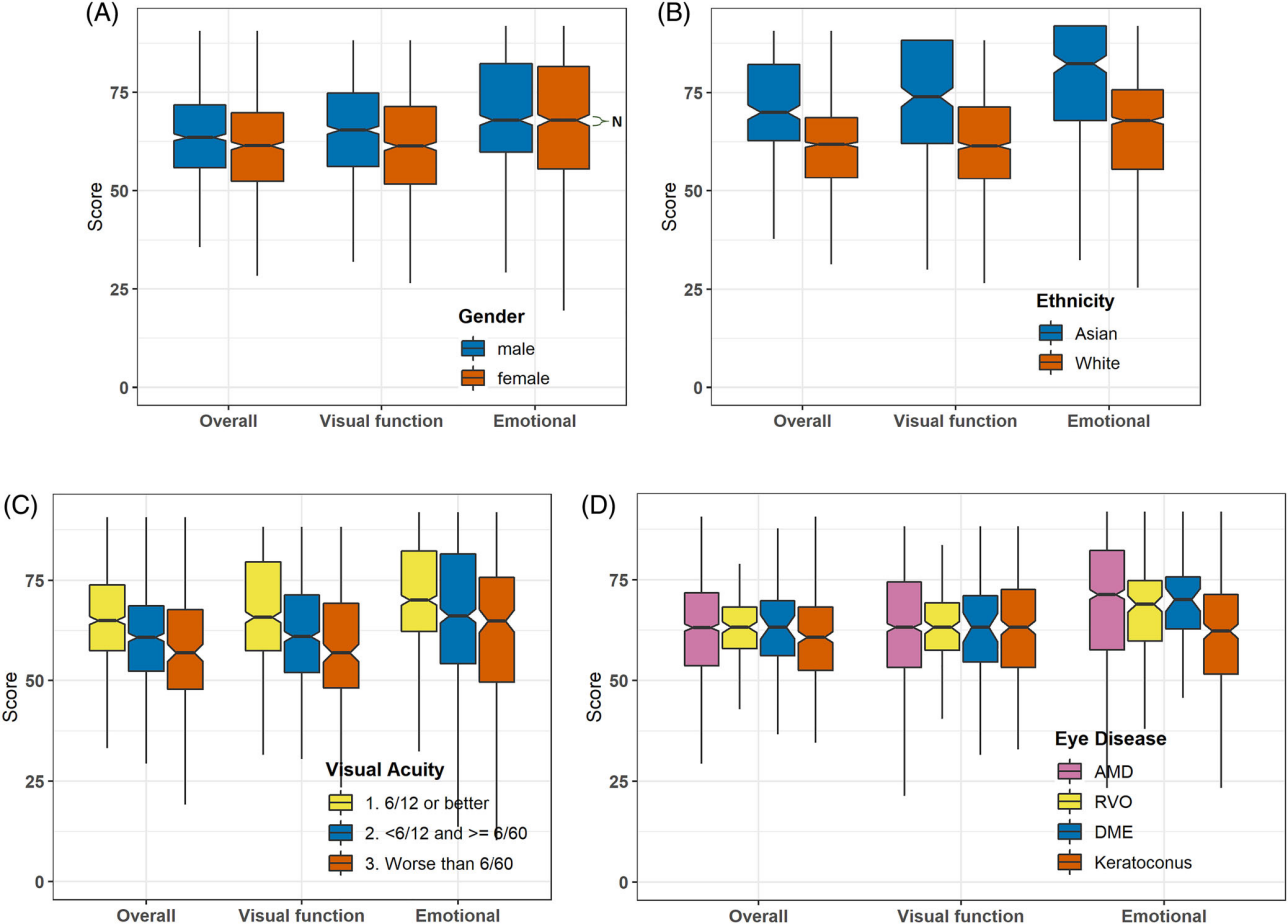
Groupwise comparison of the IVI scores. (A) Gender; Note, N = Notch, 95% confidence interval of the median. (B) Ethnicity. (C) Visual acuity group. (D) Eye disease. The original logit scale was re‐scaled using the UScale command on Winsteps such that the IVI scores were between 0 and 100, and the item mean was placed at 50 units, for convenience

The female patients had lower Overall (61.7 vs. 64.7 units), VF (62.3 vs. 66.0 units) and EM (65.9 vs. 68.3 units) IVI scores than male patients (all *p* < 0.05; Figure 2A). With regards to ethnicity, Asian patients had better IVI scores than Whites/Caucasians or others: Overall (Asian 71.3 units, white 62.0 units, others 57.3 units; both *p* < 0.001); VF (Asian 72.4 units, White 62.7 units, Others 58.9 units; both *p* < 0.001); and EM (Asian 76.9 units, White 66.0 units, Others 59.3 units; both *p* < 0.001; Figure 2B).

The patients with worse visual acuity had lower scores for Overall (worse than 6/60, 58.9 units; ≤6/12 and ≥6/60, 61.4 units; better than 6/12, 66.2 units; all pairwise *p* < 0.001), VF (worse than 6/60, 58.8 units; ≤6/12 and ≥6/60, 62.0 units, better than 6/12, 67.8 units; all pairwise *p* < 0.001), and EM (worse than 6/60, 62.9 units; ≤6/12 and ≥6/60, 65.4 units; better than 6/12, 69.9 units; all pairwise *p* < 0.001). The IVI scales scores (Overall, VF and EM) were positively correlated with visual acuity in the better or worse eye (all *p* < 0.05, Table 4). However, the strength of correlations were low (Pearson's *r*, 0.24–0.39).^41^ The strength of correlations were similar for the better (Overall 0.35, VF 0.39, EM 0.24) and worse eye (Overall 0.31, VF 0.33 and EM 0.25). Correlations with visual acuity were stronger for VF than for EM subscales.

**TABLE 4:** Impact of Vision Impairment (IVI) scores by eye diseases and their relationship with visual acuity

Eye conditions	Overall		Visual function		Emotional	
	Crude	Adjusted	Crude	Adjusted	Crude	Adjusted
Eye conditions						
Keratoconus	61.0	48.2	63.6	47.7	61.0	50.8
AMD	63.9	67.6	64.3	68.9	68.8	71.8
RVO	63.5	64.6	64.4	65.9	68.4	69.2
DME	62.3	58.8	63.3	59.4	66.7	63.1
Pearson's correlation						
VA—better eye	0.35*		0.39*		0.24*	
VA—worse eye	0.31*		0.33*		0.25*	

*Note*: AMD, age‐related macular degeneration; DME, diabetic macular edema; RVO, Retinal vein occlusion; VA, visual acuity. Adjusted values were calculated using multiple regression controlling for age, gender and baseline visual acuity. *p < 0.05.

#### 3.2.1 Comparative impact of eye diseases on QoL

The keratoconus patients had the worst crude (unadjusted) Overall score (mean, 61.0 units) compared to DME (62.3 units), RVO (63.5 units) and AMD (63.9 units) patients, however, only the difference between keratoconus and AMD patients was statistically significant (Tukey HSD, *p* = 0.003; others, *p* > 0.05). Statistically significant differences were observed on multiple regression adjusting for age, gender and baseline visual acuity. The keratoconus patients had the worst Overall score (adjusted mean: 48.2 units followed by DME 58.8 units, RVO 64.6 units and AMD 67.6 units; all pairwise differences *p* < 0.05).

The differences in mean crude VF scores between the diseases were not significant (keratoconus 63.6 units, DME 63.3 units, AMD 64.3 units, RVO 64.4 units; ANOVA, *p* = 0.998). After controlling for age, gender and baseline visual acuity, keratoconus patients had the worst VF score (adjusted mean, 47.7 units) followed by DME (59.4 units), RVO (65.9 units) and AMD (68.9 units), respectively. All pairwise differences between diseases were statistically significant (all *p* < 0.05).

Keratoconus patients had the worst mean crude EM scores (keratoconus 61.0 units vs. DME 66.7 units, RVO 68.4 units and AMD 68.8 units; all pairwise *p* < 0.05). The differences remained statistically significant after controlling for age, gender and baseline visual acuity (adjusted mean: keratoconus 50.8 units vs. DME 63.1 units, RVO 69.2 units and AMD 71.8 units; all *p* < 0.05). The difference between DME and AMD scores was statistically significant (*p* = 0.008). However, the differences between DME and RVO, and AMD and RVO were not statistically significant (both *p* > 0.05).

Disease‐specific sub‐group analysis in detail is presented in Appendix S2. The IVI was able to distinguish QoL differences within disease groups. For example, the keratoconus patients with severe disease had the worst Overall, VF and EM scores followed by moderate and mild disease (all *p* < 0.05). Similarly, among the AMD patients grouped by anigiolesion type, the Idiopathic Polypoidal Choroidal Vasculopathy (IPCV) group had better QoL status than Occult, Classic, Retinal Angiomatous Proliferation (RAP) and Disciform scar groups (all *p* < 0.05). The patients with visual acuity worse than 6/60 had the worst Overall, VF and EM scores followed by the patients with visual acuity <6/12 and ≥6/60, and with 6/12 or better in each disease group.

## 4 DISCUSSION

This large‐scale registry study explored the real‐world QoL impact of four common anterior and posterior eye diseases. Rasch analysis demonstrated that the Overall, Visual function and Emotional scales of the IVI had robust psychometric properties. When the IVI scales were used as outcome measures, the keratoconus patients were found to have the worst overall, visual function and emotional IVI scores. Among retinal conditions, in general, DME patients had the worst IVI scores followed by RVO and AMD patients. We observed weak but statistically significant correlations between the IVI scores and visual acuity.

The Overall, VF and EM scales of the IVI questionnaire had robust psychometric properties including well‐functioning response categories, measurement precision, fit statistics and unidimensionality. Targeting, a sample‐dependent characteristic, was suboptimal with fewer items available for low‐impacted people. This is understandable given that the IVI was developed in a population with vision impairment targeted to higher impacted people.^27^ Targeting may impact measurement precision.^36^ Despite this, the measurement precision was above the required value (person separation index >2; person reliability >0.80) for each scale. Overall, high‐psychometric qualities, a minimum respondent burden with uniform item‐wording, clear and consistent response options, and an optimum length of the questionnaire makes it a useful tool for use in clinics and research. Similar psychometric properties in other studies demonstrated the measurement invariance (same measurement properties across various situations) of the IVI scales which is a fundamental property of measurement.^7^, ^27^, ^42^ Uniquely the findings from this study provide clinicians confidence in using the IVI for the real‐time QoL evaluation in routine clinical practice; most published studies have been generally limited to research environments.

We found some demographic groups had worse QoL scores. Identifying vulnerable populations is important for targeted resource allocation to strengthen health care in the community. Several studies have presented the gender inequality of eye disease burden with women disproportionally affected^43^, ^44^; this study highlights a similar gender disparity in the QoL impact of eye diseases. The female participants had lower Overall, VF and EM scores. The reason for this difference could be several complex factors. For example, a study on diabetes reported female patients to have less satisfaction and poorer perception of their health status and poorer coping skills.^45^ Inter‐disease differences in Overall, VF and EM scores even after adjusting for visual acuity show that strategies to address the functional and emotional impact of eye diseases may require disease‐specific efforts beyond visual rehabilitation. While the prevalence and extent of vision impairment from keratoconus are much less than other conditions such as AMD, and keratoconus may not lead to blindness especially in high‐resource settings, this study findings signify that its impact on QoL may be disproportionate to vision loss. Keratoconus typically affects students and people in financially active years, whereas retinal diseases such as AMD primarily have an onset in old age. While the patients with keratoconus may have good visual acuity, the quality of vision may be poor. Keratoconus is chronic and progressive in nature, contact lens wear or corneal transplantation to achieve improvements in vision may be problematic and have QoL impacts. There is little comparative evidence on the QoL impact of various ophthalmic conditions. In 2004, Kymes *et al* showed that keratoconus patients had similar National Eye Institute Visual Function Questionnaire (NEI‐VFQ) scores in most QoL domains to severe AMD patients.^46^ Whereas, a German study found no statistically significant differences in IVI scores between various ocular conditions after controlling for visual acuity, perhaps due to low‐sample sizes in each sub‐group.^7^

We found worse QoL scores in people with worse visual acuity which has been similarly reported in various settings.^7^, ^47^ Statistically significant but weak strength of the correlations between Overall, VF and EM scores and visual acuity indicated that the standard clinical measures such as visual acuity alone cannot fully explain the impact of eye disease on a patient's life. In particular, a higher correlation would be expected between visual acuity and visual functioning. The weak correlations observed in the current study utilising real‐world data indicated that achieving good visual acuity is not sufficient to establish a good functional vision. Measuring contrast sensitivity, glare and other measures of visual function could be equally important. Overall, our findings highlight the need to measure the PROs including visual function and emotional impact of eye diseases in research and routine clinical practice to complement clinical measures.

The correlations of IVI scores with the better eye and worse eye were similar, in agreement with a study from Finger *et al* which highlighted the significant contribution of the worse eye in visual functioning, emotional well‐being and QoL.^48^ A person's QoL may be substantially reduced even when the better‐seeing eye has a normal or near‐normal vision.

In addition to the psychometric properties, this study also adds evidence of known‐group validity and concurrent validity of the IVI questionnaire.^42^ The Overall, VF and EM IVI scales could differentiate clinically significant groups (known‐group validity) identifying both inter‐group differences (e.g., difference in emotional status between eye diseases) as well as within‐group differences (e.g., difference in emotional status between keratoconus severity groups). Similarly, higher Overall, VF and EM scores implying better QoL status positively correlated with visual acuity in logMAR letters (concurrent validity). As expected, we found that visual acuity correlated more with Visual function than with the Emotional scores.

The study had several strengths. Our large cohort of routine clinical practice data from multiple international sites likely reflects the QoL impact of eye diseases in the real world.^29^, ^30^, ^31^, ^32^ This study has confirmed that the use of IVI in SSR modules is appropriate, and misguided or false conclusions are not derived from the future IVI use in the registry. Beyond the SSR, the findings have provided valuable information on the psychometric quality and validity of the IVI to clinicians and ophthalmic‐PRO researchers, particularly for measuring the real‐world impact from patients' perspectives. This study may serve as a benchmark for future studies exploring QoL in eye diseases in SSR and beyond.

The use of Rasch analysis, which is based on scientific measurement principles, to analyse the IVI data was another strength of this study.^13^, ^33^ Most of the existing evidence on the QoL impact of eye disease is using poor quality measures based on classical test theory (CTT) that use summary scoring. The summary scoring method is based on fallacious assumptions. The scale scores in the summary scoring method are calculated by summing and averaging ordinal data obtained using Likert scales. Each item gets an equal weight for calculating an overall score. For example, for a PRO measure consisting of items on ‘day driving’ and ‘night driving’, equal weights are given to these items, while we know that driving at night is more difficult than driving during the day. Similarly, an equal distance between adjacent response categories is assumed. Assuming the Likert‐scale data as continuous data is erroneous.^35^, ^37^, ^49^ Modern psychometric methods such as Rasch analysis are based on scientific principles and address the limitations of the CTT. Appropriate PRO measure with good psychometric quality is essential to ensure PRO‐research leads to meaningful and valid results.

The QoL is a complex construct confounded by various demographic and clinical factors. This was partly accounted for by using statistical modelling. It is important to note that In the multivariable analysis, we have calculated age‐adjusted QoL scores for eye diseases in line with the common practice.^7^, ^10^, ^21^, ^22^, ^47^ Cautious interpretation is required as age can in fact be an intermediate factor, for example, part of the causal pathway for AMD. Also, while the IVI scales were useful to study QoL differences in disease‐specific sub‐groups (Appendix S2), it should be noted that condition‐specific PRO measures may be more appropriate for a detailed disease‐specific QoL evaluation. While the study sample represents a real‐world clinical population, it may not truly represent the disease populations in the community, therefore, further warrants cautious interpretation.

In conclusion, this study has established the IVI as a robust PRO measure to evaluate QoL in routine clinical practice and improved our understanding of the QoL impacts of eye diseases highlighting the differences by demographic and clinical groups. The QoL impacts of eye diseases are not explained alone by visual acuity as indicated by low strengths of correlations. The relative impact of eye diseases on QoL will inform public health planning and resource prioritisation efforts for reducing the QoL impact of eye diseases around the world, with a goal of reducing the burden of eye diseases in individuals and communities. While cross‐sectional studies such as this allowed the evaluation of associations, future longitudinal studies using the IVI may determine the causative relationship between variables and the influence of treatment such as cross‐linking or anti‐VEGF injections on the QoL impact of eye diseases. Data derived may also assist the development of technologies and treatments able to reduce the impact on QoL of people with eye diseases.

## CONFLICT OF INTEREST

The authors declare no conflict of interest.

## Supporting information

Appendix S1 Detailed Rasch analysis. Appendix S2 Detailed characteristics of the participants and subgroup analysis by each eye disease.

**Figure S1** S1.1: Category probability curves for the Visual Function (first group) items (1 to 13 and 16 to 20).
**Figure S1.2**: Category probability curves for the Visual Function (second group) items (14 and 15).
**Figure S1.3**: Category probability curves for the Emotional items (21–28).
**Figure S1.4**: Person‐Item map for the IVI‐ Visual Function scale.
**Figure S1.5**: Person‐Item map for the IVI‐ Emotional scale.
