# Supplementary material for: Quality of life impact of eye diseases: a Save Sight Registries study
Source: Clin Exp Ophthalmol. 2022 Feb 7;50(4):386–97. doi: 10.1111/ceo.14050 (PMC9303885; doi:10.1111/ceo.14050)
Supplement: Supplementary file 1 — Appendix S1 Detailed Rasch analysis. Appendix S2 Detailed characteristics of the participants and subgroup analysis by each eye disease. [file CEO-50-386-s001.docx]

# Supporting information: Appendices

**List of Appendices:**

Appendix S1. Detailed Rasch analysis

Appendix S2. Detailed characteristics of the participants and subgroup analysis by each eye disease

**List of Supplementary tables and figures:**

**Supplementary Tables:**

Table S1.1. Category structure statistics for the Visual Function (first group) items (1 - 13 and 16 - 20)

Table S1.2. Category structure statistics for the Visual Function (second group) items (14 and 15)

Table S1.3. Category structure statistics for the Emotional items (21 to 28)

Table S1.4. Rasch parameters of the Overall, Visual Function and Emotional IVI scales

Table S1.5. Item measures and fit statistics for the IVI – Overall scale

Table S1.6. Item measures and fit statistics for the IVI – Visual Function scale

Table S1.7. Item measures and fit statistics for the IVI – Emotional scale

Table S2.1. Demographic and clinical characteristics of the keratoconus patients

Table S2.2. Demographic and clinical characteristics of the AMD patients

Table S2.3. Demographic and clinical characteristics of the RVO patients

Table S2.4. Demographic and clinical characteristics of the DME patients

**Supplementary Figures:**

Figure S1.1. Category probability curves for the Visual Function (first group) items(1 - 13 and 16 - 20)

Figure S1.2. Category probability curves for the Visual Function (second group) items (14 and 15)

Figure S1.3. Category probability curves for the Emotional items (21 to 28)

Figure S1.4 Person-Item map for the IVI- Visual Function scale

Figure S1.5 Person-Item map for the IVI- Emotional scale

# Appendix S1. Detailed Rasch analysis

The original IVI questionnaire had 28 items. Items 1 to 15 start with “In the past month, how much has your eyesight **interfered** with the following activities”, items 16 to 20 start with “In the past month, how often has your eyesight made you **concerned** or worried about the following:” and items 21-28 start with “Think about how your eyesight has made you **feel** in the past month”. Each item has 4- or 5-point response options with an additional option ‘Don’t do this for other reasons’. The responses were initially coded as 0, Not at all; 1, A little; 2, A fair amount and 3, A lot. For this study, the responses were reverse-coded as 0, A lot; 1, A fair amount; 2, A little and 3, Not at all as shown in tables S1.1 – S1.3, with lower scores for higher impact; i.e. better scores indicated better quality of life status. Category structure statistics of the three groups of items for VF (2 groups) and EM (1 group) are given below. The rating scales were scaled 7X with item means placed at 50 units.

**Table S1.1. Category structure statistics for the Visual Function (first group) items (1 to 13 and 16 to 20)**

| Category | Category count | Category % | Infit MnSq | Outfit MnSq | Threshold calibration | Category measure |
| --- | --- | --- | --- | --- | --- | --- |
| 0. A lot | 1,722 | 7 | 1.22 | 1.32 | None | (-15.57)^*^ |
| 1. A fair amount | 2,630 | 11 | 0.93 | 0.85 | -6.09 | -4.52 |
| 2. A little | 5,140 | 21 | 0.97 | 0.75 | -0.59 | 4.34 |
| 3. Not at all | 14,804 | 61 | 1.02 | 1.05 | 6.67 | (15.86) |
| Missing data^#^ | 3,694 | 13 |  |  |  |  |

Note**:** MnSq = mean square; ^*^As the extremes are at infinity, estimated values in parenthesis correspond to 0.25 score points away from the extremes. ^#^Missing data = Missing data+ Don’t do this for other reasons

**Table S1.2. Category structure statistics for the Visual Function (second group) items (14 and 15)**

| Category | Category count | Category % | Infit MnSq | Outfit MnSq | Threshold calibration | Category measure |
| --- | --- | --- | --- | --- | --- | --- |
| 0. A lot | 577 | 21 | 0.93 | 0.88 | None | (-14.01)^*^ |
| 1. A fair amount | 830 | 30 | 0.93 | 0.84 | -5.37 | 0.00 |
| 2. Not at all | 1,321 | 48 | 1.07 | 1.14 | +5.37 | (14.01) |
| Missing data^#^ | 382 | 12 |  |  |  |  |

Note: MnSq = mean square; *As the extremes are at infinity, estimated values in parenthesis correspond to 0.25 score points away from the extremes. #Missing data = Missing data+ Don’t do this for other reasons

**Table S1.3. Category structure statistics for the Emotional items (21 to 28)**

| Category | Category count | Category % | Infit MnSq | Outfit MnSq | Threshold calibration | Category measure |
| --- | --- | --- | --- | --- | --- | --- |
| 0. A lot | 1,014 | 9 | 1.28 | 1.32 | None | (-21.09)^*^ |
| 1. A fair amount | 1,295 | 11 | 0.94 | 0.95 | -12.83 | -7.57 |
| 2. A little | 3,033 | 26 | 0.92 | 0.75 | -2.19 | 6.62 |
| 3. Not at all | 6,471 | 55 | 1.06 | 1.04 | 14.56 | (22.71) |
| Missing data^#^ | 635 | 5 |  |  |  |  |

Note: MnSq = mean square; *As the extremes are at infinity, estimated values in parenthesis correspond to 0.25 score points away from the extremes. #Missing data = Missing data+ Don’t do this for other reasons

The thresholds and the category measures were ordered (**Tables S1.1 – S1.3**, **Figures S1.1 – S1.3**). The category fit statistics were satisfactory. The frequency count for the first category (A lot) was low suggesting fewer participants in the lowest visual function or emotional status (i.e. highest impact) category. However, the frequency count for the category 1 was much higher than the minimum number of responses required for a stable threshold calibration.^1^

**Figure S1.1. Category probability curves for the Visual Function (first group) items (1 to 13 and 16 to 20)**

Note: red, A lot; blue, A fair amount; pink, A little; black, Not at all

**Figure S1.2. Category probability curves for the Visual Function (second group) items (14 and 15)**

Note: red, A lot; blue, A fair amount; pink, Not at all

**Figure S1.3. Category probability curves for the Emotional items (21 to 28)**

Note: red, A lot; blue, A fair amount; pink, A little; black, Not at all

The Overall IVI scale was essentially unidimensional based on PCA variance explained by the measure, ratio of the variance explained by the items to the variance explained by the first contrast, correlation between the first and second clusters, and good fit statistics. The eigen value of the first contrast (3.4, variance explained: 5.3%) suggested the possibility of forming subscales (**Table S1.4**). A scale with a large no of items may contain subscales which increases the utility. A cluster of 7 EM items had PCA standardised residual loadings >0.40 logits, and EM21 loaded 0.34. Thus, all EM items (EM20 – EM28) had high loadings. A separate Rasch analysis with these 8 items formed the Emotional scale (**Table S1.4**). The remaining 20 items formed the Visual Function scale. The Eigenvalue of both VF and EM scales were less than 3.0. The variance explained by the measure was higher for EM (68.0%) than VF (57.5%).

The Overall IVI scale had good fit statistics **(Table S1.4)** after person weighting three erratic responses for EM25. Person weighting was done such that persons with erratic responses (residuals ≥ |4|) were weighted 0, so that they did not influence the fit statistics or measures of other persons or items. Person weighting did not affect dimensionality computations. Similarly, EM scale had good fit statistics after person-weighting 6 erratic responses for EM21. The VF scale also had excellent fit statistics.

All IVI scales (Overall, VF and EM) had satisfactory measurement precision (PSI >2.0) and no notable DIF by age, gender, visual acuity, and eye disease. Measurement precision for VF and EM scales were similar (PSI, VF 2.09 vs EM 2.14).

Person-item maps (**Figures 1.4 and 1.5**) show the relative location of persons and items in a common linear scale. Higher person measures represented a lesser impact from eye disease (higher quality of life). Similarly, the higher item measures represented the most impactful items (QoL issues) for these patients (**Tables S1.5 – S1.7**). For example, the reading items were the most impactful VF items and concern about eyesight getting worse was the most impactful EM issue for this sample. The items were satisfactorily spread along the continuum, particularly in the middle spectrum. However, the targeting, which is a sample-dependent parameter, was poor for all scales based on the difference between mean item and person locations.

**Table S1.4. Rasch parameters of the Overall, Visual Function and Emotional IVI scales**

| Parameters | Rasch model expectations | Overall | Visual Function (VF) | Emotional (EM) |
| --- | --- | --- | --- | --- |
| Disordered thresholds |  | No | No | No |
| No. of items (Ni) / No of persons (Np) |  | Ni = 28 / Np = 1,557 | Ni = 20 / Np = 1,557 | Ni = 8 / Np = 1,557 |
| PSI (person reliability) | >2.0 (>0.80) | 2.54 (0.87) | 2.07 (0.81) | 2.12 (0.82) |
| ISI (item reliability) | >3.0 (>0.90) | 13.66 (0.99) | 12.63 (0.99) | 20.55 (1.0) |
| PCA, variance by the first factor | >50% | 56.9% | 57.3% | 68.1% |
| PCA, eigen-value for 1^st^ contrast (% unexplained variance) / Pearson’s (P) and disattenuated (D) correlation between 1^st^ and 2^nd^ item clusters | < 3.0 (< 5.0%) | ***3.4*** (5.3%) / P 0.7, D 1.0 | 2.4 (5.2%) / P 0.7, D 1.0 | 1.6 (6.5%) / P 0.7, D 1.0 |
| PCA, % raw variance explained by items | - | 12.5 % | 21.7 % | 18.3 % |
| Item infit (MnSq) | <1.5 | ***-*** | ***-*** | ***EM21: 1.35*** |
| Item outfit (MnSq) | <1.5 | ***EM25: 1.37*** | ***-*** | ***-*** |
| Item measures, range (logits scaled 7X) | - | 41.34 to 59.76 | 43.27 to 60.65 | 37.10 to 64.17 |
| Targeting, difference between person & item means (logits scaled 7X) | <7.0 | ***13.23*** | ***14.17*** | ***17.16*** |
| Items with PCA standardised residual loadings > 0.4 |  | 7 EM items (EM25, EM26, EM24, EM22, EM27, EM28, EM23) + EM21 loaded 0.36 | 4 items close to 0.40 (VF18, VF11, VF13, VF16; Concern items) | 2 items (EM25 and EM26; Concern items) |

Note: Values in bold-italic font represent poor fit to the Rasch model. ISI, Item separation index; IVI, Impact of Vision Impairment; MnSq, Mean square; PCA, Principal component analysis; PSI, Person separation index

**Figure S1.4 Person-Item map for the IVI- Visual Function scale**

Note: Persons are located in the left, with their abilities in the latent traits from low (at the bottom) to high (at the top). Items are placed at the right side with their difficulty level of the latent trait from low (bottom) to high (top). M = Mean, S = one standard deviation from the mean, T = two standard deviations from the mean

**Figure S1.5 Person-Item map for the IVI- Emotional scale**

Note: Persons are located in the left, with their abilities in the latent traits from low (at the bottom) to high (at the top). Items are placed at the right side with their difficulty level of the latent trait from low (bottom) to high (top). M = Mean, S = one standard deviation from the mean, T = two standard deviations from the mean

**Table S1.5. Item measures and fit statistics for the IVI – Overall scale**

| SN | Item No | Item | Item measure | SE for Item measure | Infit MnSq | Outfit MnSq |
| --- | --- | --- | --- | --- | --- | --- |
| 1 | 14 | Reading ordinary size print | 59.76 | 0.32 | 0.99 | 1.06 |
| 2 | 25 | Worried about your eyesight getting worse | 59.62 | 0.26 | 1.24 | 1.37 |
| 3 | 8 | Reading labels or instructions on medicines | 57.78 | 0.26 | 1.14 | 1.24 |
| 4 | 22 | Felt frustrated or annoyed | 55.28 | 0.27 | 1.00 | 1.04 |
| 5 | 1 | Ability to see and enjoy TV | 53.81 | 0.31 | 1.04 | 1.14 |
| 6 | 11 | Made you go carefully to avoid falling or tripping | 53.68 | 0.27 | 1.17 | 1.21 |
| 7 | 28 | Interfered with your life in general | 53.14 | 0.28 | 0.73 | 0.71 |
| 8 | 13 | Going down steps, stairs, or curbs | 53.03 | 0.31 | 1.08 | 1.11 |
| 9 | 15 | Getting information that you need | 52.79 | 0.39 | 0.98 | 0.80 |
| 10 | 26 | Concerned or worried about coping with everyday life | 52.52 | 0.28 | 1.09 | 1.12 |
| 11 | 3 | Shopping - Finding what you want and paying for it | 50.8 | 0.33 | 1.03 | 0.83 |
| 12 | 19 | Stopped you doing the things you want to do | 50.76 | 0.32 | 0.90 | 0.83 |
| 13 | 5 | Recognising or meeting people | 49.94 | 0.29 | 1.11 | 0.94 |
| 14 | 2 | Taking part in recreational activities such as bowling, walking or golf | 49.52 | 0.37 | 1.19 | 1.15 |
| 15 | 10 | Getting about outdoors | 49.28 | 0.34 | 0.87 | 0.75 |
| 16 | 12 | Interfered with Travelling or using transport | 48.96 | 0.37 | 0.98 | 0.69 |
| 17 | 9 | Operating household appliances and the telephone | 48.59 | 0.3 | 0.99 | 0.88 |
| 18 | 20 | Needed help from other people | 48.37 | 0.3 | 0.89 | 0.88 |
| 19 | 24 | Felt sad or low | 48.3 | 0.35 | 1.12 | 0.97 |
| 20 | 7 | Opening packaging | 47.73 | 0.35 | 1.15 | 0.89 |
| 21 | 18 | Your general safety when out of your home | 46.96 | 0.31 | 0.86 | 0.76 |
| 23 | 27 | Felt like a nuisance or a burden | 46.4 | 0.32 | 1.15 | 1.07 |
| 22 | 6 | Generally looking after your appearance | 46.29 | 0.36 | 1.10 | 0.89 |
| 24 | 4 | Visiting friends or family | 44.78 | 0.39 | 1.24 | 0.77 |
| 25 | 17 | Spilling or breaking things | 43.73 | 0.35 | 0.93 | 0.83 |
| 26 | 16 | Your general safety at home | 43.67 | 0.35 | 0.97 | 0.75 |
| 27 | 21 | Felt embarrassed | 43.18 | 0.4 | 1.19 | 0.86 |
| 28 | 23 | Felt lonely or isolated | 41.34 | 0.38 | 1.21 | 0.71 |

Note. IVI, Impact of Vision Impairment; MnSq, Mean square; SE, Standard error

**Table S1.6. Item measures and fit statistics for the IVI – Visual Function scale**

| SN | Item No | Item | Item measure | SE for Item measure | Infit MnSq | Outfit MnSq |
| --- | --- | --- | --- | --- | --- | --- |
| 1 | 14 | Reading ordinary size print | 60.65 | 0.34 | 1.03 | 1.10 |
| 2 | 8 | Reading labels or instructions on medicines | 58.37 | 0.27 | 1.11 | 1.16 |
| 4 | 1 | Ability to see and enjoy TV | 53.95 | 0.32 | 1.05 | 1.22 |
| 3 | 11 | Made you go carefully to avoid falling or tripping | 53.93 | 0.28 | 1.13 | 1.19 |
| 5 | 13 | Going down steps, stairs, or curbs | 53.16 | 0.32 | 1.03 | 1.01 |
| 6 | 15 | Getting information that you need | 52.94 | 0.41 | 1.05 | 0.94 |
| 7 | 3 | Shopping - Finding what you want and paying for it | 50.8 | 0.34 | 0.93 | 0.83 |
| 8 | 19 | Stopped you doing the things you want to do | 50.73 | 0.33 | 1.05 | 1.11 |
| 9 | 5 | Recognising or meeting people | 49.88 | 0.3 | 1.07 | 0.96 |
| 10 | 2 | Taking part in recreational activities such as bowling, walking or golf | 49.47 | 0.38 | 1.16 | 1.23 |
| 11 | 10 | Getting about outdoors | 49.12 | 0.35 | 0.84 | 0.77 |
| 12 | 12 | Interfered with Travelling or using transport | 48.93 | 0.38 | 0.99 | 0.75 |
| 13 | 9 | Operating household appliances and the telephone | 48.51 | 0.31 | 0.88 | 0.78 |
| 14 | 20 | Needed help from other people | 48.26 | 0.31 | 1.02 | 1.06 |
| 15 | 7 | Opening packaging | 47.58 | 0.36 | 1.02 | 0.84 |
| 16 | 18 | Your general safety when out of your home | 46.72 | 0.32 | 0.90 | 0.84 |
| 17 | 6 | Generally looking after your appearance | 45.98 | 0.37 | 1.05 | 0.97 |
| 18 | 4 | Visiting friends or family | 44.47 | 0.4 | 1.17 | 0.75 |
| 19 | 17 | Spilling or breaking things | 43.29 | 0.36 | 1.00 | 0.86 |
| 20 | 16 | Your general safety at home | 43.27 | 0.36 | 1.02 | 0.83 |

Note. IVI, Impact of Vision Impairment; MnSq, Mean square; SE, Standard error

**Table S1.7. Item measures and fit statistics for the IVI – Emotional scale**

| SN | Item No | Item | Item measure | SE for Item measure | Infit MnSq | Outfit MnSq |
| --- | --- | --- | --- | --- | --- | --- |
| 1 | 25 | Worried about your eyesight getting worse | 64.17 | 0.31 | 0.97 | 1.02 |
| 2 | 22 | Felt frustrated or annoyed | 57.85 | 0.33 | 0.97 | 0.97 |
| 3 | 28 | Interfered with your life in general | 54.66 | 0.34 | 0.84 | 0.86 |
| 4 | 26 | Concerned or worried about coping with everyday life | 53.81 | 0.34 | 0.96 | 0.94 |
| 5 | 24 | Felt sad or low | 47.72 | 0.42 | 1.01 | 0.88 |
| 6 | 27 | Felt like a nuisance or a burden | 44.75 | 0.39 | 1.15 | 0.99 |
| 7 | 21 | Felt embarrassed | 39.94 | 0.5 | 1.36 | 0.96 |
| 8 | 23 | Felt lonely or isolated | 37.1 | 0.47 | 1.28 | 0.78 |

Note. IVI, Impact of Vision Impairment; MnSq, Mean square; SE, Standard error

# Appendix S2. Characteristics of the participants and subgroup QoL analysis by each eye disease

### **2.1 Keratoconus**

Kmax was chosen to grade the keratoconus severity as it is directly associated with the pathophysiology of keratoconus. Kmax is one of the most commonly used real-world parameter for detecting and grading keratoconus although more complex systems such Amsler-Krumeich classification and ABCD grading systems are available which consider multiple parameters.^2, 3^ Keratoconus severity was classified based on the Kmax in the better eye into the following; mild (Kmax < 48 D), moderate (Kmax 48–55 D), and severe (Kmax > 55 D).^4, 5^

**Table S2.1. Demographic and clinical characteristics of the keratoconus patients**

| Characteristics |  | | | |
| --- | --- | --- | --- | --- |
| N | 307 | | | |
| **Age**, years |  | | | |
| Mean (SD)  Min, max | 28.08 (10.9)  11, 76 | | | |
| **Gender**, n (%) |  | | | |
| Male  Female | 206 (67.1)  101 (32.9) | | | |
| **Country of residence**, n (%) | | | | |
| Australia | 306 (99.7) | | | |
| **Ethnicity**, n (%) |  | | | |
| White or Caucasian  Asian  Others  Unspecified | 206 (60.6)  22 (7.2)  28 (9.1)  71 (23.1) | | | |
| **Mode of refractive correction at presentation, n (%)** | | | | |
| Spectacles  Contact lenses  Unaided | 186 (60.6)  10 (3.3)  111 (36.2) | | | |
| **Mean visual acuity** (SD) logMAR letters | 75.3 (14.1) | | | |
| **Mean Kmax** (SD), D | 52.3 (7.3) | |  | |
| **Mean K2** (SD), D | 47.6 (4.5) | |  | |
| **Mean MCT** (SD), µm | 477.1 (46.0) | |  | |
| **Mean IVI scores by disease subgroups** | | | | |
|  | **Overall** | **Visual Function** | | **Emotional** |
| **Mean IVI scores by disease-severity** | |  | |  |
| Mild (N = 92)  Moderate (N = 115)  Severe (N = 77) | 65.6  61.1  56.7 | 69.4  63.4  59.0 | | 66.0  61.0  56.0 |
| **Mean IVI scores by treatment status** | | | | |
| CXL (N = 44)  No CXL (N = 263) | 62.6  60.7 | 65.5  63.3 | | 61.4  60.9 |
| **Mean IVI scores by visual acuity** | | | | |
| 6/12 or better (N = 242)  <6/12 and >=6/60 (N = 59)  Worse than 6/60 (N = 6) | 63.1  53.5  43.6 | 66.3  54.7  41.9 | | 63.2  52.9  48.1 |
| **Mean IVI scores (all)** | 61.0 | 63.6 | | 61.0 |

Note: CXL, Crosslinking; D, Dioptre Kmax, Maximum simulated keratometry; K2, Steep keratometry; Max, Maximum, Min = Minimum; MCT, Minimum corneal thickness; Q, Quartile; SD, standard deviation

The correlation of Overall, VF, and EM scale score with age were weak and negative(Pearson r: Overall -0.08, VF -0.09, EM -0.001), indicating worse visual function status with increasing age. However, none of them were statistically significant (all p > 0.05).

Severe keratoconus cases had lower mean Overall, VF and EM scores followed by moderate and mild keratoconus cases (**Table S2.1**). The difference between each pair was statistically significant (Tukey adjusted HSD; all p <0.05) except the emotional scores between moderate and severe groups (p = 0.121). The patients with corneal collagen cross-linking had better IVI scores . However, the differences were not statistically significant (all p >0.05).

Patients with visual acuity worse than 6/60 had the worst IVI scores followed by the patients with visual acuity <6/12 and >=6/60, and 6/12 or better, respectively (all pairwise p <0.05 except for the difference between patients with visual acuity worse than 6/60 and <6/12 and >=6/60 [p = 0.79]).

### **2.2 Age-related Macular Degeneration**

The lesion types were classified as Occult, Classic, Retinal Angiomatous Proliferation (RAP), Idiopathic Polypoidal Choroidal Vasculopathy (IPCV), Juxtapapillary, and Disciform scar.

**Table S2.2. Demographic and clinical characteristics of the AMD patients**

| Characteristics |  | | |
| --- | --- | --- | --- |
| N | 1049 | | |
| **Age**, years |  | | |
| Mean (SD)  Min, max | 79.3 (8.6)  50, 100 | | |
| **Gender**, n (%) |  | | |
| Male  Female | 467 (44.5)  582 (55.5) | | |
| **Country of residence**, n (%) |  | | |
| Australia  Singapore  Others | 798 (76.1)  212 (20.2))  39 (3.7) | | |
| **Ethnicity**, n (%) |  | | |
| White or Caucasian  Non-white  Unspecified | 796 (75.9)  244 (23.3)  9 (0.9) | | |
| **Mean Visual acuity** (SD) LogMAR letters | 56.2 (21.2) | |  |
| **Mean IOP** (SD) mm Hg | 14.3 (3.5) | |  |
| **Mean IVI scores by disease subgroups** | | | |
|  | **Overall** | **Visual Function** | **Emotional** |
| **Lesion type** |  |  |  |
| Type 1 – Occult (N = 544)  Type 2 - Classic (N = 171)  Type 3 - RAP (N = 31)  IPCV (N = 136)  Juxtapapillary (N = 10)  Disciform scar (N = 13) | 62.7  60.4  66.3  73.2  62.1  62.2 | 63.0  60.7  65.2  75.0  63.0  59.6 | 67.8  63.5  72.8  78.2  67.8  70.4 |
| **Geographic atrophy** |  |  |  |
| No (N = 388)  Subfoveal (N = 36)  Extrafoveal (N = 63) | 68.8  66.0  62.4 | 69.8  65.3  62.1 | 74.1  71.6  69.3 |
| **Mean IVI scores by visual acuity** |  |  |  |
| 6/12 or better (N = 362)  <6/12 and >=6/60 (N =498)  Worse than 6/60 (N = 189) | 68.4  62.2  59.5 | 69.2  62.7  59.3 | 74.3  66.6  63.7 |
| **Treatment status** |  |  |  |
| Treated (N = 1100)  Not treated (N = 67) | 63.9  63.4 | 64.464.0 | 68.8  67.9 |
| **Mean IVI scores (all)** | **63.9** | **64.3** | **68.8** |

Note: IPCV, Idiopathic Polypoidal Choroidal Vasculopathy; RAP, Retinal Angiomatous Proliferation

The correlation of Overall, VF and EM scale scores with age were weak but statistically significant (Spearman’s r: Overall: -0.23, VF -0.25 and EM -0.15; all p<0.05), indicating worse QoL status with increasing age.

The IPCV group had higher mean Overall scores than others (in decreasing order of Overall score: Type 3, Type 1, Disciform, Juxtapapillary, Type 2; and for VF scores: Type 3, Type 1, Juxtapapillary, Type 2, Disciform [**Table S2.2**]; Tukey HSD; all p<0.05 except for the differences between IPCV and Juxtapapillary groups [both p > 0.05]). The differences between other groups were not statistically significant for both Overall and VF scales (all p >0.05). Similarly, the IPCV group had the highest mean EM score followed by Type 3, Disciform scar, Juxtapapillary, Type 1, and Type 2. On pair-wise evaluation, the difference in IPCV and Type 1, and IPCV and Type 2 were statistically significant (both p < 0.05). All other pairwise differences were not statistically significant (all p >0.05).

Patients with extrafoveal geographical atrophy had the worst scores followed by the patients with sub-foveal and no geographical atrophy. On pairwise evaluation, only the differences between extrafoveal geographical atrophy and no geographical atrophy were statistically significant (Tukey HSD, all p <0.05).

Patients with visual acuity worse than 6/60 had the worst IVI scores followed by the patients with visual acuity <6/12 and >=6/60, and 6/12 or better, respectively (all pairwise p <0.05 except the difference in EM score between the groups with visual acuity worse than 6/60 and with <6/12 and >=6/60 [p = 0.141]).

Patients who received treatment (anti-VEGF injection, laser therapy) had higher Overall, VF and EM scores than those who were treatment naïve. However, the differences were not statistically significant (all p >0.05).

### **2.3 Retinal Vein Occlusion**

The RVO types was classified as branched-retinal vein occlusion (BRVO), central retinal vein occlusion (CRVO) and hemi-retinal vein occlusion (HRVO). All cases had a history of receiving treatment (anti-VEGF / laser).

**Table S2.3. Demographic and clinical characteristics of the RVO patients**

| Characteristics | |  | | | |
| --- | --- | --- | --- | --- | --- |
| N | | 148 | | | |
| **Age**, years | |  | | | |
| Mean (SD)  Min, max | | 73.1 (9.7)  41, 96 | | | |
| **Gender**, n (%) | |  | | | |
| Male  Female | | 84 (56.8)  64 (43.2) | | | |
| **Country of residence**, n (%) | |  | | | |
| Australia | | 147 (99.3) | | | |
| **Ethnicity**, n (%) | |  | | | |
| White or Caucasian  Others | | 136 (91.9)  12 (8.1) | | | |
| **Visual acuity**, mean (SD) logMAR letters | | 60.1 (21.4) | | | |
| **Mean IVI scores by disease sub-groups** | | | | |  |
|  | **Overall** | | **Visual Function** | **Emotional** |  |
| **Type** |  | |  |  |  |
| BRVO (N = 45)  CRVO (N = 95)  HRVO (N = 8) | 64.5  62.9  65.2 | | 65.4  63.8  65.9 | 69.8  67.2  75.4 |  |
| **Mean IVI scores by visual acuity** |  | |  |  |  |
| 1. 6/12 or better (N = 59)  2. <6/12 and >=6/60 (N =68)  3. Worse than 6/60 (N = 21) | 66.4  62.7  58.0 | | 67.6  63.3  59.1 | 70.7  68.8  60.7 |  |
| **Mean IVI scores (all)** | **63.5** | | **64.4** | **68.4** |  |

**Note:** BRVO, branched-retinal vein occlusion; CRVO, central retinal vein occlusion (CRVO); HRVO, hemi-retinal vein occlusion

The correlation of Overall and VF scale scores with age was weak but statistically significant (Spearman’s r: Overall: -0.18, p = 0.026; and VF -0.25, p = 0.002) indicating worse overall QoL status and worse visual functioning with increasing age. The correlation between EM scores and age was not statistically significant (Spearman’s r: -0.07, p = 0.368)

The CRVO group had the worst scores followed by BRVO and HRVO. However, on pairwise evaluations, none of the differences were statistically significant (all p > 0.05).

Patients with visual acuity worse than 6/60 had the worst IVI scores followed by the patients with visual acuity <6/12 and >=6/60, and 6/12 or better, respectively (one-way ANOVA; all p < 0.05) Tukey HSD test revealed statistical significance for difference between category 1 and 2, and 1 and 3 for the Overall and VF scales (all p <0.05). The differences of Overall and VF scores between category 2 and 3 were not statistically significant (Overall p = 0.101; VF p = 0.205). For EM scale, the difference between category 1 and 3 and 2 and 3 were statistically significant (both p <0.05). However, the difference in EM scores between category 1 and 2 was not significant (p = 0.701)

### **2.4 Diabetic Macular Edema**

The types and severity of diabetic retinopathy was graded as mild non-proliferative diabetic retinopathy (NPDR), moderate NPDR, severe NPDR and proliferative diabetic retinopathy (PDR).^6^

**Table S2.4. Demographic and clinical characteristics of the DME patients**

| Characteristics |  | | |  |
| --- | --- | --- | --- | --- |
| N | 53 | | |  |
| **Age,** years |  | | |  |
| Mean (SD)  Min, max | 64.9 (9.6)  46, 86 | | |  |
| **Age at DME diagnosis**, years |  | | |  |
| Mean (SD)  Min, max | 46.0 (15.3)  0, 70 | | |  |
| **Gender**, n (%) |  | | |  |
| Male  Female | 34 (64.2)  19 (35.8) | | |  |
| **Country of residence**, n (%) |  | | |  |
| Australia  Others | 44 (83.0)  9 (17.0) | | |  |
| **Ethnicity**, n (%) |  | | |  |
| White or Caucasian  Others | 43 (81.1)  10 (18.9) | | |  |
| **History of DME treatment**, n (%) | 47 (88.7) | |  |  |
| **Visual acuity** logMAR letters, mean (SD) | 73.8 (12.9) | |  |  |
| **CST** µm, mean (SD) | 349.0 (124.3) | |  |  |
| **Mean IVI scores by DME sub-groups** | | | | |
|  | **Overall** | **Visual Function** | **Emotional** | |
| **Diabetes type** |  |  |  | |
| Type I (N = 22)  Type II (N = 31) | 58.6  64.9 | 60.2  65.6 | 60.4  71.3 | |
| **DR grades** |  |  |  | |
| Mild NPDR (N = 14)  Moderate NPDR (N = 20)  Severe NPDR (N = 11)  PDR (N = 8; including 4 treated cases) | 65.3  60.1  58.0  68.3 | 66.6  60.4  59.1  70.9 | 71.5  64.8  59.3  73.5 | |
| **DME activity** |  |  |  | |
| Centre involving CSME (N = 42)  Non-centre involving CSME (N = 4)  No CSME (N = 7) | 62.0  63.2  63.5 | 63.1  64.8  63.8 | 66.1  65.5  71.4 | |
| **Mean IVI scores by visual acuity** |  |  |  | |
| 6/12 or better (N = 42)  <6/12 and >=6/60 (N = 10)  Worse than 6/60 (N = 1) | 64.1  56.1  45.2 | 65.2  57.5  45.4 | 69.5  57.7  41.7 | |
| **Treatment status** |  |  |  | |
| Treated (N = 47)  Not treated (N = 6) | 62.3  62.1 | 63.3  63.9 | 67.0  64.8 | |
| **Mean IVI scores (all)** | 62.3 | 63.3 | 64.4 | |

Note: CSME, clinically significant macular edema; CST, central subfield thickness; DME, diabetic macular edema; logMAR, logarithm of minimal angle of resolution; NPDR, non-proliferative diabetic retinopathy; PDR, Proliferative diabetic retinopathy

The correlation of Overall, VF and EM scale scores with age was not statistically significant (Spearman’s r: Overall: -0.12, VF -0.22 and EM 0.09; all p>0.05).

Type 1 diabetes had worse emotional score than Type 2 diabetes (0.018). While Type 1 patients had worse Overall and VF scores than Type 2 diabetes, the differences were not statistically significant (p = 0.062 and 0.148 respectively).

Less sample size (n = 53) limited us from doing detailed subgroup analysis and reaching strong conclusions. For NPDR, scores worsened as the severity worsened. Half of the PDR cases (n = 4) had received treatment, and had slightly better IVI scores. All pairwise differences between the DME groups were not statistically significant (Tukey adjusted HSD, all p >0.05). Similarly, the differences in IVI scores between the CSME activity types (none; centre involving; non-centre involving) were not statistically significant (all p > 0.05).

Patients with visual acuity worse than 6/60 had the worst IVI scores followed by the patients with visual acuity <6/12 and >=6/60, and 6/12 or better, respectively (one-way ANOVA; all p = 0.07, 0.11 and 0.05 for Overall, VF and EM). None of the pairwise differences were statistically significant (all p >0.05) perhaps due to low sample size in two groups.

Patients who received treatment (anti-VEGF injection, laser therapy) had slightly better Overall, VF and EM scores to those who were treatment naïve, however, the differences were not statistically significant (all p >0.05). Strong conclusions could not be derived due to a small sample size in the treatment naïve group.

**Appendix References:**

1. Bond T, Fox CM. Rasch modeling applied: Rating scale design. In: Bond T, Fox CM, editors. Applying the Rasch model: fundamental measurement in the human sciences. 3^rd^ ed. New York: Routledge; 2015. p. 245-64.

2. Tur VM, MacGregor C, Jayaswal R*, et al.* A review of keratoconus: diagnosis, pathophysiology, and genetics. *Surv Ophthalmol*. 2017;62(6):770-83.

3. Saunier V, Mercier AE, Gaboriau T*, et al.* Vision-related quality-of-life and dependency in French keratoconus patients: Impact study. *J Cataract Refract Surg*. 2017;43(12):1582-90.

4. Tan JC, Nguyen V, Fenwick E*, et al.* Vision-related quality-of-life in keratoconus: a Save Sight Keratoconus Registry study. *Cornea*. 2019;38(5):600-5.

5. Kandel H, Pesudovs K, Ferdi A*, et al.* Psychometric properties of the Keratoconus Outcomes Research Questionnaire (KORQ): a Save Sight Keratoconus Registry study. *Cornea*. 2020;39(3):303-10.

6. Bhandari S, Nguyen V, Fraser-Bell S*, et al.* Ranibizumab or Aflibercept for diabetic macular edema: comparison of 1-year outcomes from the fight retinal blindness! Registry. *Ophthalmology*. 2020;127(5):608-15.
