# Supplementary figures and images for: Quality of life impact of eye diseases: a Save Sight Registries study
Source: Clin Exp Ophthalmol. 2022 Feb 7;50(4):386–97. doi: 10.1111/ceo.14050 (PMC9303885; doi:10.1111/ceo.14050)

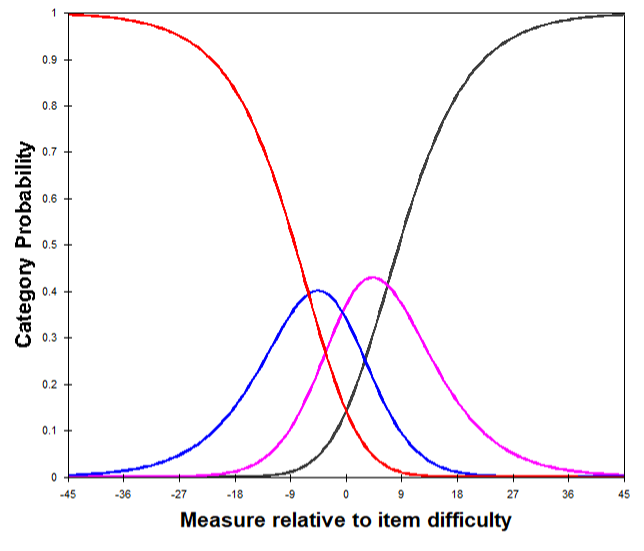

Supplement: Supplementary file 2 — Figure S1 S1.1: Category probability curves for the Visual Function (first group) items (1 to 13 and 16 to 20). Figure S1.2: Category probability curves for the Visual Function (second group) items (14 and 15). Figure S1.3: Category probability curves for the Emotional items (21–28). Figure S1.4: Person‐Item map for the IVI‐ Visual Function scale. Figure S1.5: Person‐Item map for the IVI‐ Emotional scale. [file CEO-50-386-s002.zip › ceo14050-sup-0002-FigureS1-HKedited/CEO_14050_CEO-21-11-1054 Figure S1.1.tif]

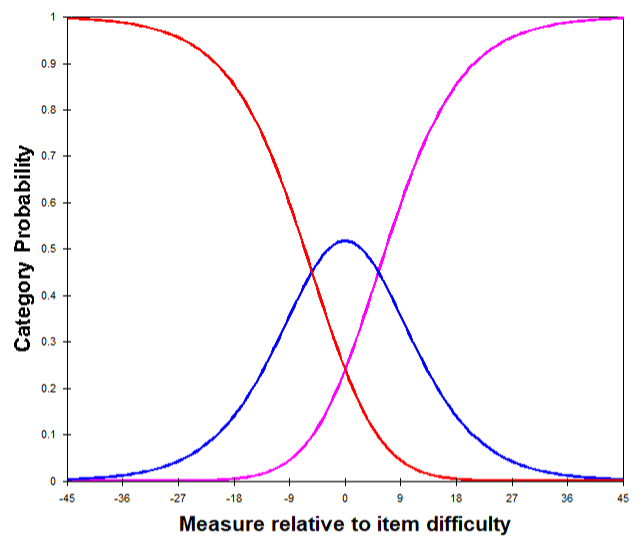

Supplement: Supplementary file 2 — Figure S1 S1.1: Category probability curves for the Visual Function (first group) items (1 to 13 and 16 to 20). Figure S1.2: Category probability curves for the Visual Function (second group) items (14 and 15). Figure S1.3: Category probability curves for the Emotional items (21–28). Figure S1.4: Person‐Item map for the IVI‐ Visual Function scale. Figure S1.5: Person‐Item map for the IVI‐ Emotional scale. [file CEO-50-386-s002.zip › ceo14050-sup-0002-FigureS1-HKedited/CEO_14050_CEO-21-11-1054 Figure S1.2.tif]

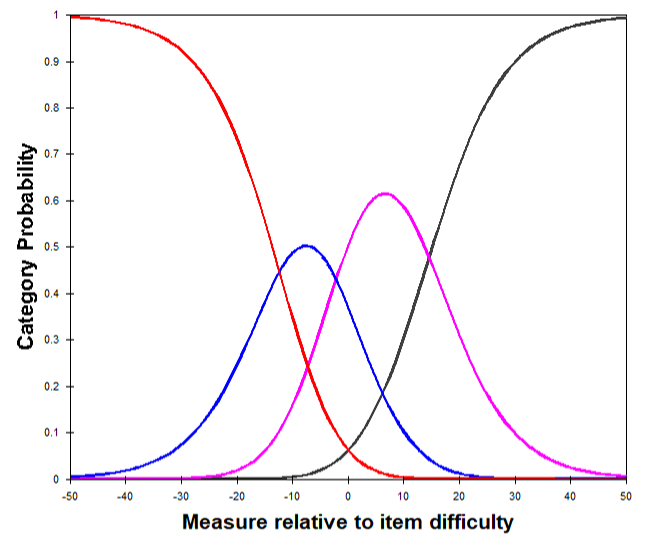

Supplement: Supplementary file 2 — Figure S1 S1.1: Category probability curves for the Visual Function (first group) items (1 to 13 and 16 to 20). Figure S1.2: Category probability curves for the Visual Function (second group) items (14 and 15). Figure S1.3: Category probability curves for the Emotional items (21–28). Figure S1.4: Person‐Item map for the IVI‐ Visual Function scale. Figure S1.5: Person‐Item map for the IVI‐ Emotional scale. [file CEO-50-386-s002.zip › ceo14050-sup-0002-FigureS1-HKedited/CEO_14050_CEO-21-11-1054 Figure S1.3.tif]

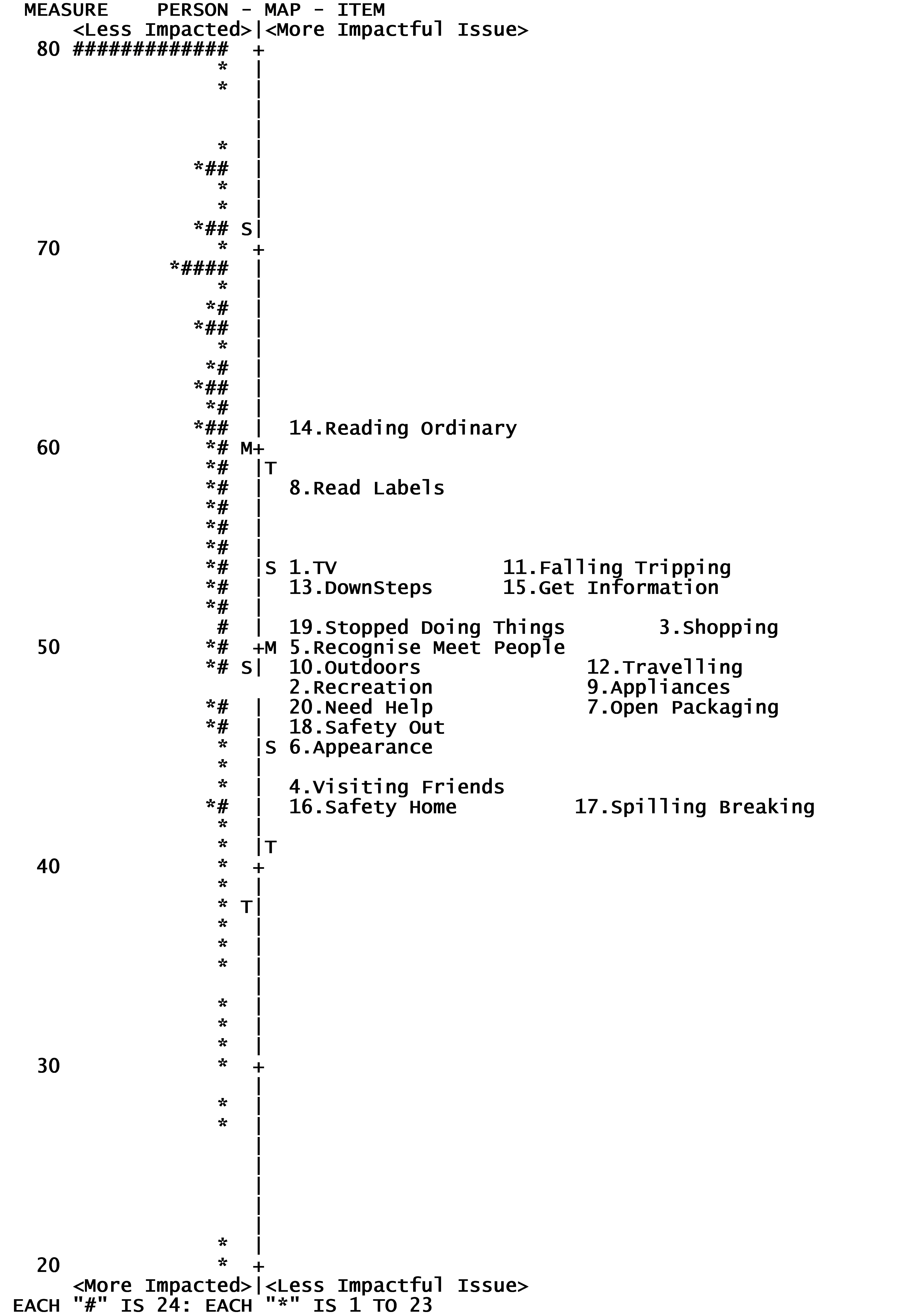

Supplement: Supplementary file 2 — Figure S1 S1.1: Category probability curves for the Visual Function (first group) items (1 to 13 and 16 to 20). Figure S1.2: Category probability curves for the Visual Function (second group) items (14 and 15). Figure S1.3: Category probability curves for the Emotional items (21–28). Figure S1.4: Person‐Item map for the IVI‐ Visual Function scale. Figure S1.5: Person‐Item map for the IVI‐ Emotional scale. [file CEO-50-386-s002.zip › ceo14050-sup-0002-FigureS1-HKedited/CEO_14050_CEO-21-11-1054 Figure S1.4.tif]
